# Thermogravimetric Analysis of Moisture in Natural and Thermally Treated Clay Materials

**DOI:** 10.3390/ma17102231

**Published:** 2024-05-09

**Authors:** Giulia Lo Dico, Lorenzo Lisuzzo, Verónica Carcelén, Giuseppe Cavallaro, Maciej Haranczyk

**Affiliations:** 1 IMDEA Materials Institute, C/Eric Kandel 2, Getafe, 28906 Madrid, Spain; glodico@tolsa.com; 2 Tolsa Group, Carretera de Madrid a Rivas Jarama, 35, 28031 Madrid, Spain; vcarcelen@tolsa.com; 3 Department of Materials Science and Engineering, Universidad Carlos III de Madrid, Avda. de la Unive sidad, 30, Leganés, 28911 Madrid, Spain; 4 Department of Physics and Chemistry, University of Palermo, Viale delle Scienze, pad. 17, 90128 Palermo, Italy; lorenzo.lisuzzo@unipa.it

**Keywords:** microporous clays, MTGA, dehydration, adsorption, activation energy

## Abstract

Clays are a class of porous materials; their surfaces are naturally covered by moisture. Weak thermal treatment may be considered practical to remove the water molecules, changing the surface properties and making the micro- and/or mesoporosities accessible to interact with other molecules. Herein, a modulated thermogravimetric analysis (MTGA) study of the moisture behavior on the structures of five, both fibrous and laminar, clay minerals is reported. The effect of the thermal treatment at 150 °C, which provokes the release of weakly adsorbed water molecules, was also investigated. The activation energies for the removal of the adsorbed water (Ea) were calculated, and they were found to be higher, namely, from 160 to 190 kJ mol^−1^, for fibrous clay minerals compared to lamellar structures, ranging in this latter case from 80 to 100 kJ mol^−1^. The thermal treatment enhances the rehydration in Na-montmorillonite, stevensite, and sepiolite structures with a decrease in the energy required to remove it, while Ea increases significantly in palygorskite (from 164 to 273 kJ mol^−1^). As a proof of concept, the MTGA results are statistically correlated, together with a full characterization of the physico-chemical properties of the five clay minerals, with the adsorption of two molecules, i.e., aflatoxin B1 (AFB1) and β-carotene. Herein, the amount of adsorbed molecules ranges from 12 to 97% for the former and from 22 to 35% for the latter, depending on the particular clay. The Ea was correlated with AFB1 adsorption with a Spearman score of −0.9. When the adsorbed water is forcibly removed, e.g., under vacuum conditions and high temperatures, the structure becomes the most important, decreasing the Spearman score between β-carotene and Ea to −0.6.

## 1. Introduction

Clays, due to their low cost, biocompatibility, and environmentally friendly nature [1], have been extensively utilized as adsorbents for a wide range of applications. They are effective in adsorbing toxic molecules [2], organic pollutants [3], and pigment impurities [4,5,6], and serve as nanocontainers for bioactive molecules [7,8] and nanocarriers for drug delivery systems [9,10]. Clay nanoparticles are suitable as fillers of polymers to obtain nanocomposites useful for packaging purposes [11,12] as well as building materials [13,14]. Moreover, nanoclays can be employed in cosmetic formulations [15,16,17,18] and the conservation of Cultural Heritage [19]. The crystal structure of clay minerals consists of a repetition of layers consisting of either one octahedral sheet and one tetrahedral sheet (1:1-type clay minerals) or one octahedral sheet sandwiched between two tetrahedral sheets (2:1-type clay minerals). This structural arrangement results in diverse morphologies, such as fibrous structures (e.g., sepiolites and palygorskites) or laminar structures (e.g., montmontmorillonites and stevensites. The physicochemical properties of clays are correlated with the particle size and their deposits. Smectites also exhibit a high cation exchange capacity (CEC) correlated with their swelling properties [20]. Natural clays contain moisture due to water adsorption on both external and internal surfaces. The moisture content of raw clays also varies since water can be reversibly adsorbed and desorbed during natural dehydration and rehydration processes depending on the environmental conditions. The weakest adsorbed water molecules are externally adsorbed water and the water present in micro- and mesopores, which can be eliminated by heating between 40 and 200 °C for sepiolites and palygorskites or under vacuum treatment [21,22]. The presence of the water molecules plays a crucial role in the adsorption properties of clays as it influences the accessibility of micro- and mesopores to other molecules [23,24]. For instance, higher moisture content enhances the interlayer space of swelling montmorillonites, altering the morphological properties and making the microporous structure suitable for trapping organic molecules [25]. On the other hand, the presence of water layers on the clay micro- and mesoporous surfaces may hinder productive interactions with guest molecules [26,27]. The competition between guest molecules and water during adsorption depends on their hydrophilic nature [28]. Mild thermal treatment can remove moisture without significantly modifying the clay structure, and it has been found to enhance the uptake of organic guest molecules [27,29]. The coordination water, which is bonded to octahedral Mg^2+^ at the edges (Mg-OH_2_), is important for preserving the crystal structure of clays, and its loss at temperatures over 300 °C leads to a reduction in crystallinity [30,31]. Thus, thermal treatment at high temperatures causes profound changes in clay morphology, significantly reducing the accessible surface area of the micro- and mesoporous structures, and as a consequence, the adsorption properties [29,32,33]. The implementation of vacuum pumping is an effective strategy to remove the water molecules, releasing the clay porosity and enhancing the loading rate [34]. Lisuzzo et al. [35] investigated the water confinement within the cavity of halloysite by Knudsen thermogravimetry and described the mechanism of halloysite loading. Relative to bulk water, the fraction present on the porosities displays higher vapor pressure and faster evaporation due to the Gibbs–Thomson effect. As water evaporates, guest molecules precipitate in the cavity, while vacuum pumping aids solvent migration to the halloysite lumen. The dehydration mechanism of clay minerals was investigated with various approaches, also by modulated thermogravimetric analysis TGA measurement (MTGA), extracting a fast calculation of the activation energy (Ea) associated with the removal of the water from the material surfaces [36,37,38,39,40,41,42]. In addition to moisture, the adsorption efficiency of clays depends on their morphology, porosity, and the polar properties of the clay surfaces, which can vary depending on the natural environment and mining sources [43,44]. Adsorption mechanisms hinge on surface properties and adsorbate characteristics. Electrostatic forces play a primary role in cation and charged molecule adsorption on clays, and are strongly correlated with the clay zeta potential (ζ) [45]. On the other hand, various other mechanisms, including van der Waals forces, hydrophobic interaction, and hydrogen bonding, can occur [45,46,47]. Natural substitutions in the 2:1 lattice structures and vacancies impart them with a cation exchange capacity and charge the layers of those clay minerals with a layer charge of 0.2–0.6 per formula unit (pfu). Clays such as montmorillonites, sepiolites, and stevensites exhibit pH-dependent behavior, including isoelectric points, based on their specific chemical structure and morphology [48,49,50]. Consequently, selective adsorption is achievable by manipulating the electrostatic forces between the clay surface and the target molecule [51]. This contribution presents a comprehensive study of the behavior of adsorbed water in a series of 2:1 phyllosilicate structures with different morphologies. The role of moisture in natural clays and thermally treated clay is investigated using MTGA analysis to calculate the kinetic parameters associated with water removal from the clay surfaces. As a proof of concept, the activation energy (Ea) is correlated with other physicochemical properties with the adsorption activity of natural clays in two diverse applications: mycotoxin aflatoxin B1 (AFB1) and β-carotene mitigation. The adsorption experiments try to simulate real-life application scenarios and are tailored to emulate the conditions of the gastrointestinal tract for AFB1 adsorption, while employing vacuum pumping and elevated temperatures to facilitate β-carotene adsorption.

## 2. Materials and Methods

### 2.1. Materials Characterization

Five samples rich in 2:1 phyllosilicates, namely, fibrous sepiolite and palygorskite, and laminar Na-montmorillonite, Ca-montmorillonite, and stevensite, were obtained from TOLSA S.A. and used without any prior treatment. The samples were milled in a Retsch ZM 300 at 18,000 rpm, with a 0.2 mm mesh. Particle size was determined by calculating the percentage of fine particles in the sample capable of passing through pores with a diameter of 45 µm. Approximately 95% of the particles possess a size < 45 µm. XRD diffractograms were recorded with a D8-ADVANCE diffractometer (Bruker, Billerica, MA, USA), using Cu Kα radiation. The voltage and current sources were set at 40 kV and 30 mA, respectively. The patterns were recorded between 2° and 70° (θ degrees) at a goniometer speed of 0.5 s per step. To estimate the phyllosilicate content, the size fraction (fine size material < 45 μm) was isolated by centrifugation and used as oriented aggregates for clay–mineral identification and semi-quantification. The semi-quantification is based on the reflectance ratios provided by Schultz (1964) [52]. Thus, the intensity of a characteristic reflection of a mineral divided by its reflectance ratio is directly proportional to the amount of mineral in the sample. Since XRD technique cannot give precise quantitative information about the amount of phyllosilicate in the sample, the calculated amounts of each phyllosilicate were determined with respect to the total content present in each raw sample, and on the basis of a wide dataset of different clay samples used as references. Chemical analysis of metal oxide content was performed using an ICP-OES Varian, Agilent 730 instrument (Santa Clara, CA, USA), and expressed in terms of weight percentage (wt%). The loss by calcination was evaluated by subjecting the samples to calcination at 1000 °C in an air atmosphere, and the resulting mass loss was determined. Textural characteristics were evaluated using physisorption isotherms measured with a Micromeritics Gemini V surface area and pore size analyzer. The isotherms were acquired after degassing the samples for 18 h at 124 °C. Nitrogen gas was employed as the adsorbate at 77 K, and the textural parameters were calculated based on the adsorption branch of the N_2_ isotherm. The specific surface area (SA) and external surface area (ESA) were determined using the Brunauer–Emmett–Teller (BET) theory. The total pore volume (Pore vol), micropore content (Micro), and main pore size (MPS) were calculated using the t-plot method [53,54,55]. These parameters provide insights into the surface area, porosity, and pore size distribution of the clays. The cation exchange capacity (CEC) was estimated by quantifying the concentration of the Cu^2+^-triethylenetetramine complex using spectrophotometry. For this purpose, an exchange solution (1 L) was prepared by dissolving 1.45 mL of triethylenetetramine (97%, Sigma-Aldrich, St. Louis, MO, USA) and 100 mL of 0.1 M copper sulfate standard solution (Sigma Aldrich) in deionized water. To evaluate the released cations (Na^+^, Ca^2+^, Mg^2+^, K^+^) from the clay structure, an exchange solution (30 mL) was added to 0.15 g of clay sample, and the suspension was vigorously stirred using a vortex agitator until no visible agglomerations were detected. Subsequently, the suspension was centrifuged for 5 min at 4500 rpm, and the supernatant solution was measured using a UV spectrophotometer (Agilent Technologies, Santa Clara, CA, USA, Cary 60 UV–vis) at 580 nm. The cations released by the clay structure (Na^+^, Ca^2+^, Mg^+2^, K^+^), in the supernatant solution, were evaluated by chemical analysis with inductively coupled plasma optical emission spectroscopy (ICP-OES) Varian, Agilent 730 (Santa Clara, CA, USA). Zeta potential (ζ) experiments were carried out using a Zetasizer NANO-ZS (Malvern Instruments, London, UK) at 25 °C. A dispersion of each sample (ca. 10^−3^ wt%) was placed in a disposable folded capillary cell and used for measurements. Water contact angle tests were performed with an optical contact angle apparatus (OCA 20, Data Physics Instruments, Filderstadt, Germany) equipped with a video measuring system having a high-resolution CCD camera and a high-performance digitizing adapter. Data acquisition was conducted by SCA 20 software (Data Physics Instruments). The clay sample was prepared by compressing the powder into a tablet using a press of high pressure. The contact angle (θ) of water in air was detected through the sessile drop method by placing a water droplet of 10 ± 0.5 mL onto the surface of the clay tablets. The measurements were conducted at 30.0 ± 0.1 °C (Figure S2 in Supplementary Materials). The morphology of the natural clays was evaluated by scanning electron microscopy (SEM) using a FE-SEM Apreo 2S LoVac- Field Emission Scanning Electron Microscope (FEG-SEM) (Thermo Fisher Scientific, Waltham, MA, USA) without pretreating the studied samples.

### 2.2. Thermal Treatment and MTGA Measurements

To investigate the role of the water at the micro- and mesoporosities of the natural clays, the samples were treated by conditioning in a desiccator, and the relative humidity was maintained at 75% using a saturated NaCl solution for 48 h at room temperature. The samples were then dried at 150 °C for 24 h to remove the adsorbed water and reconditioned under the same humidity conditions for 48 h. MTGA was performed with a Thermogravimetric Analyzer Discovery TGA550, and TRIOS software (version 5.1.1) was further used to analyze data. Measurements were carried out under the nitrogen flow of 60 cm^3^ min^−1^ for the sample and 40 cm^3^ min^−1^ for the balance. The temperature range was between 25 and 260 °C. Each sample was placed in a platinum pan and heated under the modulated temperature of 5 °C min^−1^ for 200 s. Then, a heating ramp of 2 °C min^−1^ was applied up to 260 °C. According to the technique, the modulated temperature is specified by Equation (1):

T_m_(t) = T_o_ + *a*t + Asin(2πωt) (1)

where T_o_ is the initial temperature (corresponding to the room temperature), *a* is the heating rate, ω is the frequency (the number of oscillations per second), and A is the amplitude of the modulation. The MTGA is a thermogravimetric experiment in which the temperature is rapidly modulated while its average within the time recording is kept under a given linear heating ramp [36,37]. The sinusoidal temperature oscillation of the MTGA enables the kinetics from a single experiment to be calculated, avoiding the need to run multiple TGAs [56]. The activation energy for the evaporating process of adsorbed water from both conditioned (Ea) and reconditioned (Ea (Rec)) samples was determined.

### 2.3. Adsorption Experiments on AFB1 Mycotoxin

Five reconditioned clays after thermal treatment were tested in adsorption experiments of AFB1 mycotoxin. The latter were chosen to mimic the adsorption through the gastrointestinal tract. In particular, clay and AFB1 mycotoxin were dispersed in phosphate buffers adjusted to pH 5 with final concentrations of 2 mg mL^−1^ of clay and 2 µg mL^−1^ of AFB1. The solution was incubated at 37 °C for 3 h under gently stirring and centrifugated at 8 m s^−1^ for 10 min to separate the complexed clay–mycotoxin from the free toxin. The amount of free toxin was quantified by high-performance liquid chromatography with ultraviolet detection (HPLC-UV). The adsorption is calculated against the amount of toxin in the standard according to Equation (2), where A_ft_ and A_st_ are the amounts of toxin in the supernatant solution and in the standard, respectively.

Ads(%) = 100 − (A_ft_/A_st_·100) (2)

### 2.4. Adsorption Experiments of β-Carotene

Five reconditioned clays after thermal treatment and reconditioned, as previously described, were tested in adsorption experiments of β-carotene. The clay (1.5 mg) was combined with 150 mg of crude palm oil in a glass reactor (250 mL) and kept under stirring for 30 min with controlled pressure of 60 mbar and temperature of 95 °C. Then, the clay was removed by filtration over a Buchner funnel with Filterlab 1250 with Ø porous of 10–13 µm and the filtered oil was cooled at 70 °C, and the amount of β-carotene was quantified using a Lovibond Tintometer Color Scale in a 0.25″ (10 mm) glass cell. The amount of β-carotene was measured in both crude (β-carotene_st_) and treated oil (β-carotene_free_) samples, calculating the corresponding adsorbed amount according to Equation (3).

β-carotene ads (%) = 100 − (β-carotene_free_/β-carotene_st_·100) (3)

### 2.5. Physico-Chemical Properties–Adsorption Correlation

To investigate the property–activity relationships, a correlation matrix based on Spearman’s coefficient (ρ) was calculated. The latter returns a score that is related to a non-parametric statistical dependence between the ranking of two variables, avoiding assumptions about the type of correlation (e.g., linear, exponential). The score is calculated as follows:

a. Calculate the ranks for each property, i.e., ordering the property values corresponding to the five studied clays from the smallest to the greatest and assigning an increasing integer value.
b. Calculate the difference between the ranks of each individual property (d).
c. Calculate the Spearman’s score (ρ) between each individual physico-chemical property (x) and the adsorption of AFB1 mycotoxin or β-carotene (y) by Equation (4).

(4) $$\rho_{x,y}=1-\frac{6\sum d_{i}^{2}}{5\left(5^{2}-1\right)}$$

The Spearman’s coefficient ranges between −1 and 1. Values close to 1 or −1 correspond to a positive or negative, respectively, monotonic correlation, while 0 relates to no correlation between ranks. Together with ρ, we considered the *p*-values to evaluate the statistical significance of the obtained correlation since our number of experiments is limited.

## 3. Results

### 3.1. Properties of Natural Clay Minerals

**Figure 1:**
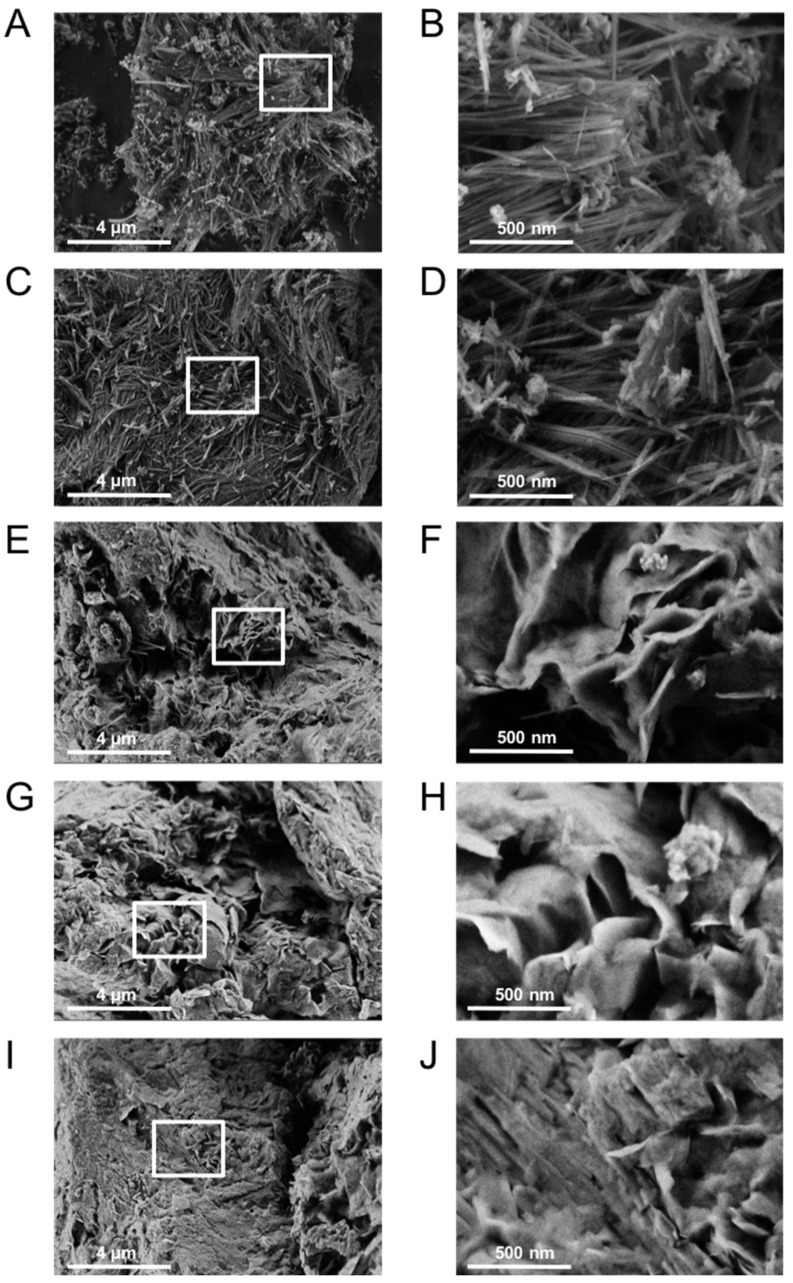
SEM micrographs of (**A**,**B**) natural palygorskite, (**C**,**D**) natural sepiolite, (**E**,**F**) natural stevensite, (**G**,**H**) Ca-montmorillonite, (**I**,**J**) natural Na-montmorillonite. Images with higher magnification on the right side correspond to the white boxes of the images on the left side.

The morphology of the fibrous clays, namely, palygorskite and sepiolite, as well as the planar clays, including stevensite, Ca-montmorillonite, and Na-montmorillonite, were observed using electron microscopy (Figure 1). The fibrous morphology of palygorskite and sepiolite (Figure 1A–D) was clearly observed, with fiber lengths ranging from 0.5 to 4 μm and thicknesses of approximately 30–50 nm. In contrast, Figure 1E–J show profiles of messily arranged lamellae with a thickness of around 10 nm. The lamellar structures of stevensite, Ca-montmorillonite, and Na-montmorillonite are associated with their swelling properties, which are advantageous for applications such as adsorption and rheological applications [2,57]. Table 1 presents a summary of the physicochemical characterization of the clays, including their total phyllosilicate content and major chemical analysis. The XRD patterns with the identified main crystalline components are reported in Figure S1 (Supplementary Materials). The clays exhibited high purity grades, with a total phyllosilicate content of at least 80%. From chemical analysis, montmorillonites possess at least 17% Al_2_O_3_, while stevensite and sepiolite have the highest amounts of MgO. The loss by calcination was found to be around 6–10% allowing use of the natural mineral sources without initial purification treatment. In addition to XRD and chemical analysis, further techniques such as N_2_ adsorption–desorption isotherms were employed to determine the specific surface area, pore volume, and pore size distribution. Porosity plays a major role in the understanding of the adsorption behavior and potential applications of clays, as reported in the literature [44,58]. The BET surface area results indicated that the fibrous clays exhibited higher surface areas compared to the laminar-shaped minerals, while the natural montmorillonites displayed the lowest surface area. These findings are in good agreement with the literature, which reports that fibrous clays typically possess a higher surface area, ca. 180 m^2^ g^−1^ for palygorskites and 300 m^2^ g^−1^ for sepiolites, whereas stevensites exhibit surface areas of ca. 70 m^2^ g^−1^ for Al-rich and 200 m^2^ g^−1^ for Mg-rich variants [44,59,60,61]. The difference in surface area can be attributed to the different morphologies and arrangements of the clay particles. In particular, under the experimental condition in which the porosity of the clays was evaluated (see Section 2), the contribution of microporosity was due to the micropores, which were located at the edges of the crystallites [1,61]. While the presence of mesopores and macropores, considered in the total pore volume and the main pore size, is assigned to the arrangement between the fibers and plates themselves [1,44]. The cation exchange capacity (CEC) of the montmorillonites was higher than that of the Mg-rich clays. On the other hand, for montmorillonites, being dioctahedral, both CEC and ζ potential are mainly controlled by the heterovalent substitutions in the structure.

### 3.2. The Thermal Treatment Effect on the Behavior of the Adsorbed Water

**Figure 2:**
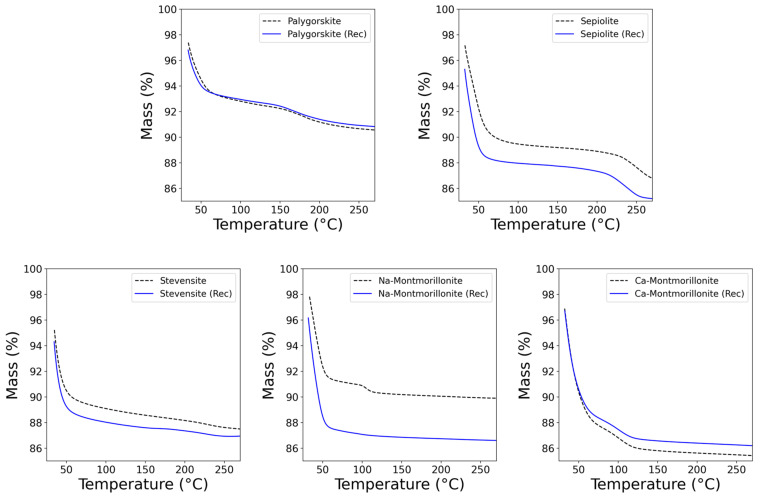
MTGA of conditioned sample under constant humidity (dotted lines) and reconditioned samples after drying overnight and 150 °C (continuous lines).

The investigation of adsorbed water behavior with thermal treatment provides insights into the dehydration mechanisms and the stability of water molecules within the clays. It is worth noting that the presence of moisture is related to the adsorption of water molecules onto the clay surfaces due to arising interactions with hydrophilic groups (e.g., -OH), which can act as active sites for the anchoring of H_2_O. By recording the MTGA profiles, it was possible to identify different temperature ranges at which water was released from the clays. The MTGA experiments allowed us to have an estimation of the average activation energy for water loss phenomena. Although differences were observed between the Ea obtained by MTGA and using multi-heating isoconversional methods, mainly due to the mathematical approximations underlying MTGA, it can provide a useful parameter for a relative comparison of the changes due to processing a given material [62]. Natural moisture occupies the mesoporosity of clays and the corresponding water molecules are removed by thermal treatment below 300 °C. From this temperature, structural OH groups also begin to be lost. These aspects are crucial since the treatment under higher temperature conditions can detrimentally affect the properties of the clays by causing a loss of crystallinity and a variation in morphological features due to the irreversible dehydration and dehydroxylation, thus making it impossible to recover the clays [30,31]. Then, MTGA profiles were recorded up to 260 °C, and they were used to investigate the behavior of the adsorbed water. Heating at 150 °C provokes the release of the adsorbed moisture, and it is expected to modify the moisture capture capacity when the dried samples are reconditioned under controlled humidity with 75 wt% saturated NaCl salt solution. The mechanism of dehydration of clays depends on the morphology and structural characteristics, e.g., type of exchangeable cations, swelling properties, and hydrophilic properties [63,64,65,66]. Figure 2 contains the MTGA profiles for the five classes of 2:1 structures. The interlayer space contains both weakly adsorbed water and water molecules strongly adsorbed to interlayer cations. The first loss at ca. 50 °C is associated with the partial removal of the moisture content and weakly/physisorbed water from the microporous and mesoporous surfaces of clays. The second loss, occurring at higher temperature, is associated with the removal of water in the microporosity of tunnels in fibrous clay and interlayer space of stevensite and montmorillonites, strongly bound to exchangeable cations. Table 2 summarizes the main parameters extracted from the MTGA profiles. Despite there being no difference in the temperature of the first loss observed after the reconditioning procedure, the moisture capture capacity was modified by the drying process. The second loss remains quite stable for non-swelling clay minerals, they not being affected by thermal treatment at 150 °C, meaning that the water uptake in the microporosity is a reversible reaction that does not affect structural and morphological properties. On the contrary, the second loss disappears from the MTGA profile of swelling Na-montmorillonite when treated at 150 °C and reconditioned. The removal of this adsorbed water is irreversible [64,66]. The Ea of conditioned fibrous clays was found higher than lamellar minerals, suggesting that the morphological differences in clay particles influence the interaction of water and clay surface in terms of the energy required to activate the process. Indeed, the removal of bound water from the mesoporosity of fibrous clays is a more energetically costly process compared to the same process occurring in lamellar clay minerals. According to the literature, indeed, the dehydration mechanism is controlled by nucleation and growth, then followed by a diffusion-controlled reaction [40,67]. The differences between the two morphologies could most likely be related to the tortuous path created by the arrangement of fibers of sepiolite or palygorskite, which hinders the removal of the water molecules [42,68,69,70,71]. After the thermal treatment is carried out, the moisture uptake is enhanced for Na-montmorillonite, stevensite, and sepiolite as observed by the increase in first loss percentages in Table 2. More interestingly, the increase in moisture capture, after drying, is accompanied by a decrease in the energy required to remove the adsorbed water from the three clays and, more effectively, from sepiolite. As a consequence, these minerals can still adsorb more water molecules, but the interaction is weaker than pristine clays before thermal treatment. On the other side, the activation energy values increased for Ca-montmorillonite and palygorskite after the drying process, despite no difference being observed in terms of moisture uptake efficiency, which remained quite stable. These results suggest that the thermal treatment promotes the adsorption of water molecules at the mineral surface making their removal harder, with particular emphasis on palygorskite.

### 3.3. Properties–Activity Correlation

**Figure 3:**
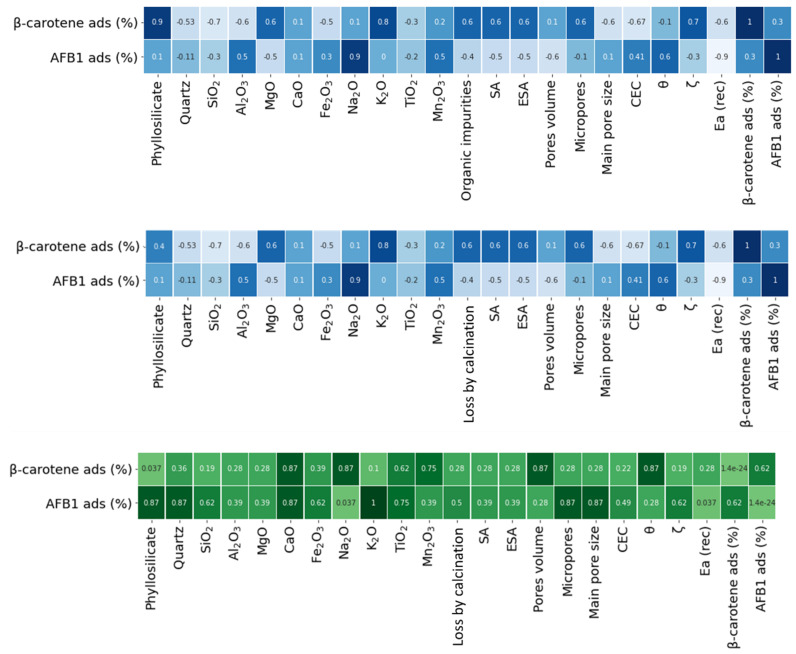
Spearman correlation coefficients (**top**, **middle**) and their associated *p*-values (**bottom**) between the adsorptive outcomes and the physico-chemical properties resumed in Table 1.

One of the main applications of natural porous clays is the removal of certain organic molecules through adsorption on the clay surfaces. Within this issue, the presence of water molecules significantly impacts the adsorption properties by affecting the accessibility of pores to other molecules. Depending on the type of clays involved, the presence of adsorbed H_2_O could hinder the absorption of organic guest molecules. Conversely, a higher moisture content can expand the interlayer space, thereby making the porous structure suitable for trapping organic molecules. Here, the mitigation of β-carotene and AFB1 mycotoxin was chosen as an example of a practical application of a clay-based adsorbent [72,73,74]. Two different experiments were performed reproducing the real adsorption process (details in Section 2). The results are shown in Table 3. The observed adsorption effectiveness of smectites is higher than that of fibrous clays, in agreement with the literature [72,75,76,77,78]. Palygorskite material removed a smaller amount of both molecules. Na-montmorillonite adsorbed more than 96% of AFB1, while stevensite was the most effective in mitigating the β-carotene content. The property–activity correlations were estimated by calculating Spearman’s coefficient (ρ), shown in Figure 3 (see Section 2 for details). In particular, ρ was calculated for each property listed in Table 1, together with the calculated Ea (Rec), and the adsorption power of Table 3. Additionally, Figure 3 displays the corresponding *p*-value for each pair of variables (green). A low ρ of 0.3 with an associated *p* of 0.62 is shown between AFB1 mycotoxin and β-carotene. The non-correlation reflects the different mechanisms involved including the different experimental setups. In fact, the adsorption experiment of β-carotene was performed at a high temperature and under vacuum condition while AFB1 was at room temperature and pressure. As expected, the adsorption of AFB1 suffers in the presence of water where it has not been removed by the temperature or the vacuum. We observed a Spearman’s correlation coefficient of −0.9 (*p* = 0.037) between Ea and the adsorption of AFB1. Ea represents the energy barrier required to displace water from a surface, allowing the adsorption of molecules like AFB1. The negative value indicates that lower Ea values correspond to higher AFB1 adsorption. Specifically, lower Ea values for water desorption suggest an increased availability of surface sites for AFB1 adsorption. Conversely, materials like palygorskite, characterized by high Ea values, imply strong water adhesion to clay particles, reducing the availability of binding sites for guest molecules, including AFB1. Moreover, the thermal treatment deteriorates the capability of palygorskite in capturing AFB1, since it increases its Ea (Rec) of 72 kJ mol^−1^, while it is effective for sepiolite structures providing a decrease of 53 kJ mol^−1^, hence favoring AFB1 adsorption with respect to the conditioned non-treated sepiolite. As further evidence of the correlation between Ea and AFB1, we performed the adsorption test of two selected untreated samples of the trioctahedral group, stevensite (small ΔE of −3 kJ mol^−1^) and sepiolite (lowest ΔE of −53 kJ mol^−1^). AFB1 adsorption of 93% for the raw stevensite and 50% for sepiolite were found. The results remark that clays, where Ea remains almost unmodified by the thermal treatment, show the same adsorption capacity, while clays like sepiolite take benefit from the thermal treatment, which, by reducing the Ea, facilitates the interaction between AFB1 and the clay surface. On the other hand, during the β-carotene mitigation experiments, the water is forcibly removed favoring the entrapping of the pigment on the clay surfaces. In this case, the decreasing of the correlation score (ρ = −0.6, *p* = 0.28) is observed, suggesting a faint competition between the guest molecule and water, it being removed by the process. The correlation between clay structure (amount of phyllosilicate) and β-carotene shows a Spearman score of 0.4 (*p* = 0.037). This observation can be interpreted considering the vacuum pumping and the high temperature promoting a good interaction between β-carotene and the clay by exposing the clay surfaces to the water. Hence, the adsorption in this case is mainly guided by the amount of active minerals. The same experiments were repeated using untreated samples and the results are shown in Table S2 (see Supplementary Materials). The observed differences with respect to the thermally treated materials remark that the clay moisture and the Ea do not significantly influence the adsorption of β-carotene due to the high temperature and the vacuum condition of the experimental process.

## 4. Conclusions

The role of water in five diverse clay structures was investigated through modulated thermogravimetry analysis (MTGA). The calculated activation energy associated with the removal of the adsorbed water (Ea) was higher in fibrous clays compared to lamellar structures. The thermal treatment at 150 °C influenced the activation energy in different ways depending on the proper morphology and structure of the 2:1 structures. In fact, the water uptake of non-swelling clay minerals was not significantly affected by the thermal treatment, suggesting a reversible reaction that does not change the structural and morphological properties. On the contrary, the thermal treatment irreversibly removes the adsorbed water in swelling Na-montmorillonite, which was lost at 98 °C in the raw material. The thermal treatment enhances the moisture uptake for Na-montmorillonite, stevensite, and sepiolite with a decrease in the required energy, whereas Ea increases in palygorskite structures. The structural, morphological, and polar properties of clays were also correlated to the adsorption of two model organic molecules, namely, aflatoxin B1 (AFB1) and β-carotene. The experiments were designed to imitate some real case applications, i.e., an additive for mycotoxin mitigation, and oil refining. Ea is the most correlated feature with AFB1 adsorption with a Spearman score of −0.9 while showing a correlation of −0.6 with β-carotene adsorption, which is mostly dependent on the phyllosilicate content. The presence of water on the clay surfaces prevents a good interaction with the target molecules, such as AFB1. When the adsorbed water is forcibly removed, for example, under vacuum condition and high temperature, the structure and electrokinetic properties become most correlated with the adsorption of organic molecules such as β-carotene.

## Figures and Tables

**Table 1:** Physico-chemical characterization of the five selected natural clays.

Property	Palygorskite	Sepiolite	Stevensite	Na-Montmorillonite	Ca-Montmorillonite
Phyllosilicate ^a^	81	96	92	88	90
Quartz ^a^	3	0	1	1	1
SiO_2_^a^	68.4	60.2	58.5	61.9	56.7
Al_2_O_3_^a^	8.2	2.0	5.4	19.3	17.3
MgO ^a^	8.6	25.7	22.2	3.2	3.8
CaO ^a^	1.5	1.0	1.5	1.4	4.7
Fe_2_O_3_^a^	3.7	0.5	1.5	4.3	5.2
Na_2_O ^a^	0.1	0.3	0.4	3.2	1.1
K_2_O ^a^	0.4	0.6	1.1	0.3	0.7
TiO_2_^a^	0.3	0.1	0.2	0.2	0.5
Mn_2_O_3_^a^	0.0	0.0	0.0	0.0	0.1
Loss by calcination ^a^	8.7	10.1	9.0	6,1	9.3
SA (m^2^ g^−1^)	150	310	248	45	63
ESA (m^2^ g^−1^)	110	152	150	28	42
Pore vol (cm^3^ g^−1^)	0.42	0.68	0.24	0.2	0.12
Micro (%)	6	12	20	5	4.7
Main pore size (Å)	97	87	37	102	113
CEC (cmol(+)/kg)	24	12	12	93	88
Na^+^ (cmol(+)/kg)	1	1	1	81	3
Ca^2+^ (cmol(+)/kg)	9	7	7	17	67
Mg^2+^ (cmol(+)/kg)	8	4	4	13	10
K^+^ (cmol(+)/kg)	1	1	1	1	1
Θ (degree)	18.4	15.6	38.1	41.8	8.3
ζ (mV)	−15	−13	−12	−26	−18

^a^ The amount is calculated as wt% with respect to the total (see Section 2).

**Table 2:** Weight loss and activation energy calculated by MTGA for conditioned sample under constant humidity and reconditioned (Rec) samples after drying overnight and 150 °C.

Sample	T (°C)	I Loss (%)	T (°C)	II Loss (%)	Ea (kJ mol^−1^)	ΔE (kJ mol^−1^)
Na_Montmorillonite	45.26	8.9	108.3	1.7	100	−13
Na_Montmorillonite (Rec)	45.16	12.7	-	0.3	87	
Ca-Montmorillonite	39.1	12.3	108	1.9	82	8
Ca-Montmorillonite (Rec)	39.1	12	106.6	2	90	
Palygorskite	38.8	7.2	180.2	2.2	164	72
Palygorskite (Rec)	37.7	7	178.2	2.2	236	
Stevensite	37.8	11.7	223.6	0.7	85	−3
Stevensite (Rec)	37.4	12.3	221	0.9	82	
Sepiolite	46.7	10.9	236.9	2.8	187	−53
Sepiolite (Rec)	45	12.2	227.4	2.8	133	

**Table 3:** β-carotene and AFB1 adsorption results for the 5 classes of minerals.

Clay Minerals	β-Carotene Ads (%) ^a^	AFB1 Ads (%) ^a^
Na-Montmorillonite (Rec)	31.5	97
Ca-Montmorillonite (Rec)	31.5	67
Palygorskite (Rec)	22	12
Stevensite (Rec)	35	91
Sepiolite (Rec)	32	63

^a^ The amount is expressed as wt% of the adsorbed molecule with respect to the total starting amount.

## Data Availability

The original contributions presented in the study are included in the article/Supplementary Materials, further inquiries can be directed to the corresponding author/s.

## Supplementary Materials

The following supporting information can be downloaded at https://www.mdpi.com/article/10.3390/ma17102231/s1, Figure S1. XRD patterns of the studied five samples. Figure S2. Images of the water droplets just after their deposition on the surface of the five clay samples. Table S1. Main crystalline components identified in the XRD patterns. Table S2. β-carotene1 adsorption results for the untreated 5 classes of minerals.

## References

[1] Bergaya F., Theng B.K.G., Lagaly G. (2006). Handbook of Clay Science.

[2] Di Gregorio M.C., De Neeff D.V., Jager A.V., Corassin C.H., Carão Á.C.D.P., De Albuquerque R., De Azevedo A.C., Oliveira C.A.F. (2014). Mineral Adsorbents for Prevention of Mycotoxins in Animal Feeds. Toxin Rev..

[3] Lambert J.F. (2018). Organic Pollutant Adsorption on Clay Minerals. Surface and Interface Chemistry of Clay Minerals.

[4] Lo Dico G., Semilia F., Milioto S., Parisi F., Cavallaro G., Inguì G., Makaremi M., Pasbakhsh P., Lazzara G. (2018). Microemulsion Encapsulated into Halloysite Nanotubes and Their Applications for Cleaning of a Marble Surface. Appl. Sci..

[5] Rožić L., Petrović S., Novaković T. (2009). β-Carotene Removal from Soybean Oil with Smectite Clay Using Central Composite Design. Russ. J. Phys. Chem. A.

[6] Sarma G.K., SenGupta S., Bhattacharyya K.G. (2011). Methylene Blue Adsorption on Natural and Modified Clays. Sep. Sci. Technol..

[7] Lo Dico G., Wicklein B., Lisuzzo L., Lazzara G., Aranda P., Ruiz-Hitzky E. (2019). Multicomponent Bionanocomposites Based on Clay Nanoarchitectures for Electrochemical Devices. Beilstein J. Nanotechnol..

[8] Lisuzzo L., Wicklein B., Lo Dico G., Lazzara G., Del Real G., Aranda P., Ruiz-Hitzky E. (2020). Functional Biohybrid Materials Based on Halloysite, Sepiolite and Cellulose Nanofibers for Health Applications. Dalton Trans..

[9] Cavallaro G., Lazzara G., Milioto S. (2023). Nanocomposites Based on Halloysite Nanotubes and Sulphated Galactan from Red Seaweed Gloiopeltis: Properties and Delivery Capacity of Sodium Diclofenac. Int. J. Biol. Macromol..

[10] Lisuzzo L., Cavallaro G., Milioto S., Lazzara G. (2024). Coating of Silk Sutures by Halloysite/Wax Pickering Emulsions for Controlled Delivery of Eosin. Appl. Clay Sci..

[11] Boccalon E., Viscusi G., Lamberti E., Fancello F., Zara S., Sassi P., Marinozzi M., Nocchetti M., Gorrasi G. (2022). Composite Films Containing Red Onion Skin Extract as Intelligent pH Indicators for Food Packaging. Appl. Surf. Sci..

[12] Bertolino V., Cavallaro G., Lazzara G., Milioto S., Parisi F. (2018). Halloysite Nanotubes Sandwiched between Chitosan Layers: Novel Bionanocomposites with Multilayer Structures. New J. Chem..

[13] Calvino M.M., Lisuzzo L., Cavallaro G., Lazzara G., Milioto S. (2022). Halloysite Based Geopolymers Filled with Wax Microparticles as Sustainable Building Materials with Enhanced Thermo-Mechanical Performances. J. Environ. Chem. Eng..

[14] Jaradat Y., Matalkah F. (2021). Olive Biomass Ash-Based Geopolymer Composite: Development and Characterisation. Adv. Appl. Ceram..

[15] Cavallaro G., Milioto S., Konnova S., Fakhrullina G., Akhatova F., Lazzara G., Fakhrullin R., Lvov Y. (2020). Halloysite/Keratin Nanocomposite for Human Hair Photoprotection Coating. ACS Appl. Mater. Interfaces.

[16] Cavallaro G., Caruso M.R., Milioto S., Fakhrullin R., Lazzara G. (2022). Keratin/Alginate Hybrid Hydrogels Filled with Halloysite Clay Nanotubes for Protective Treatment of Human Hair. Int. J. Biol. Macromol..

[17] Panchal A., Fakhrullina G., Fakhrullin R., Lvov Y. (2018). Self-Assembly of Clay Nanotubes on Hair Surface for Medical and Cosmetic Formulations. Nanoscale.

[18] Santos A.C., Panchal A., Rahman N., Pereira-Silva M., Pereira I., Veiga F., Lvov Y. (2019). Evolution of Hair Treatment and Care: Prospects of Nanotube-Based Formulations. Nanomaterials.

[19] Caruso M.R., Cavallaro G., Lazzara G., Milioto S. (2023). Pectin/Microwax Composites for Surface Coating and Protection. Mater. Lett..

[20] Teich-McGoldrick S.L., Greathouse J.A., Jové-Colón C.F., Cygan R.T. (2015). Swelling Properties of Montmorillonite and Beidellite Clay Minerals from Molecular Simulation: Comparison of Temperature, Interlayer Cation, and Charge Location Effects. J. Phys. Chem. C.

[21] Coudert F.X., Boutin A., Fuchs A.H. (2021). Open Questions on Water Confined in Nanoporous Materials. Commun. Chem..

[22] Zhou J., Lu X., Boek E.S. (2016). Confined Water in Tunnel Nanopores of Sepiolite: Insights from Molecular Simulations. Am. Mineral..

[23] Kecili R., Hussain C.M. (2018). Mechanism of Adsorption on Nanomaterials.

[24] Sadegh H., Ali G.A.M., Gupta V.K., Makhlouf A.S.H., Shahryari-ghoshekandi R., Nadagouda M.N., Sillanpää M., Megiel E. (2017). The Role of Nanomaterials as Effective Adsorbents and Their Applications in Wastewater Treatment. J. Nanostruct. Chem..

[25] Zhu J., Wang T., Zhu R., Ge F., Yuan P., He H. (2011). Expansion Characteristics of Organo Montmorillonites during the Intercalation, Aging, Drying and Rehydration Processes: Effect of Surfactant/CEC Ratio. Colloids Surf. A Physicochem. Eng. Asp..

[26] Ramírez A., Sifuentes C., Manciu F.S., Komarneni S., Pannell K.H., Chianelli R.R. (2011). The Effect of Si/Al Ratio and Moisture on an Organic/Inorganic Hybrid Material: Thioindigo/Montmorillonite. Appl. Clay Sci..

[27] Çakany Ç., Cabbar H.C. (2008). Adsorption of *p*-xylene in Dry and Moist Clay. J. Int. Environ. Appl. Sci..

[28] Hunter-Sellars E., Tee J.J., Parkin I.P., Williams D.R. (2020). Adsorption of Volatile Organic Compounds by Industrial Porous Materials: Impact of Relative Humidity. Microporous Mesoporous Mater..

[29] Balci S. (1999). Effect of Heating and Acid Pre-Treatment on Pore Size Distribution of Sepiolite. Clay Miner..

[30] Clauer N., Fallick A.E., Galán E., Pozo M., Taylor C. (2012). Varied Crystallization Conditions for Neogene Sepiolite and Associated Mg-Clays from Madrid Basin (Spain) Traced by Oxygen and Hydrogen Isotope Geochemistry. Geochim. Cosmochim. Acta.

[31] Sarıkaya Y., Önal M., Pekdemir A.D. (2020). Kinetic and Thermodynamic Approaches on Thermal Degradation of Sepiolite Crystal Using XRD-Analysis. J. Therm. Anal. Calorim..

[32] Sarı Yılmaz M., Kalpaklı Y., Pişkin S. (2013). Thermal Behavior and Dehydroxylation Kinetics of Naturally Occurring Sepiolite and Bentonite. J. Therm. Anal. Calorim..

[33] Emmerich K., Christidis G.E. (2010). Thermal Analysis in the Characterization and Processing of Industrial Minerals. Advances in the Characterization of Industrial Minerals.

[34] Abdullayev E., Lvov Y. (2011). Halloysite Clay Nanotubes for Controlled Release of Protective Agents. J. Nanosci. Nanotechnol..

[35] Lisuzzo L., Cavallaro G., Pasbakhsh P., Milioto S., Lazzara G. (2019). Why Does Vacuum Drive to the Loading of Halloysite Nanotubes? The Key Role of Water Confinement. J. Colloid Interface Sci..

[36] Blaine R.L., Hahn B.K. (1998). Obtaining Kinetic Parameters from Modulated Thermogrvimetry. J. Therm. Anal. Calorimetryand Calorim..

[37] Mamleev V., Bourbigot S. (2005). Modulated Thermogravimetry in Analysis of Decomposition Kinetics. Chem. Eng. Sci..

[38] Poinsignon C., Yvon J., Mercier R. (1982). Dehydration Energy of the Exchangeable Cations in Montmorillonite—A DTA Study. Isr. J. Chem..

[39] Girgis B.S., El-Barawy K.A., Felix N.S. (1987). Dehydration Kinetics of Some Smectites: A Thermogravimetric Study. Thermochim. Acta.

[40] Bray H.J., Redfern S.A.T. (1999). Kinetics of Dehydration of Ca-Montmorillonite. Phys. Chem. Miner..

[41] Zabat M., Van Damme H. (2000). Evaluation of the Energy Barrier for Dehydration of Homoionic (Li, Na, Cs, Mg, Ca, Ba, Alx(OH)Z+y and La)-Montmorillonite by a Differentiation Method. Clay Miner..

[42] Kuligiewicz A., Derkowski A. (2017). Tightly Bound Water in Smectites. Am. Mineral..

[43] D’Ascanio V., Greco D., Menicagli E., Santovito E., Catucci L., Logrieco A.F., Avantaggiato G. (2019). The Role of Geological Origin of Smectites and of Their Physico-Chemical Properties on Aflatoxin Adsorption. Appl. Clay Sci..

[44] Suárez M., García-Romero E. (2012). Variability of the Surface Properties of Sepiolite. Appl. Clay Sci..

[45] Zadaka D., Radian A., Mishael Y.G. (2010). Applying Zeta Potential Measurements to Characterize the Adsorption on Montmorillonite of Organic Cations as Monomers, Micelles, or Polymers. J. Colloid Interface Sci..

[46] Ewis D., Ba-Abbad M.M., Benamor A., El-Naas M.H. (2022). Adsorption of Organic Water Pollutants by Clays and Clay Minerals Composites: A Comprehensive Review. Appl. Clay Sci..

[47] Deng Y., Velázquez A.L.B., Billes F., Dixon J.B. (2010). Bonding Mechanisms between Aflatoxin B1 and Smectite. Appl. Clay Sci..

[48] Leroy P., Tournassat C., Bernard O., Devau N., Azaroual M. (2015). The Electrophoretic Mobility of Montmorillonite. Zeta Potential and Surface Conductivity Effects. J. Colloid Interface Sci..

[49] Sabah E., Mart U., Çinar M., Çelik M.S. (2007). Zeta Potentials of Sepiolite Suspensions in Concentrated Monovalent Electrolytes. Sep. Sci. Technol..

[50] Tombácz E., Szekeres M. (2004). Colloidal Behavior of Aqueous Montmorillonite Suspensions: The Specific Role of pH in the Presence of Indifferent Electrolytes. Appl. Clay Sci..

[51] Tekin N., Dinçer A., Demirbaş Ö., Alkan M. (2006). Adsorption of Cationic Polyacrylamide onto Sepiolite. J. Hazard. Mater..

[52] Schultz L.G. (1964). Quantitative Interpretation of Mineralogical Composition from X-Ray and Chemical Data for the Pierre Shale: Analytical Methods in Geochemical Investigations of the Pierre Shale.

[53] De Lange M.F., Vlugt T.J.H., Gascon J., Kapteijn F. (2014). Adsorptive Characterization of Porous Solids: Error Analysis Guides the Way. Microporous Mesoporous Mater..

[54] Thommes M., Kaneko K., Neimark A.V., Olivier J.P., Rodriguez-Reinoso F., Rouquerol J., Sing K.S.W. (2015). Physisorption of Gases, with Special Reference to the Evaluation of Surface Area and Pore Size Distribution (IUPAC Technical Report). Pure Appl. Chem..

[55] (2010). Determination of the Specific Surface Area of Solids by Gas Adsorption—BET Method.

[56] Mamleev V., Bourbigot S. (2002). Calculation of Activation Energies Using the Sinusoidally Modulated Temperature. J. Therm. Anal. Calorim..

[57] Lo Dico G., Muñoz B., Carcelén V., Haranczyk M. (2022). Data-Driven Experimental Design of Rheological Clay–Polymer Composites. Ind. Eng. Chem. Res..

[58] Zango Z.U., Garba A., Garba Z.N., Zango M.U., Usman F., Lim J. (2022). Montmorillonite for Adsorption and Catalytic Elimination of Pollutants from Wastewater: A State-of-the-Arts Review. Sustainablity.

[59] Martin de Vidales J.L., Pozo M., Alia J.M., Garcia-Navarro F., Rull F. (1991). Kerolite-Stevensite Mixed-Layers from the Madrid Basin, Central Spain. Clay Miner..

[60] Suárez M., García-Rivas J., Morales J., Lorenzo A., García-Vicente A., García-Romero E. (2022). Review and New Data on the Surface Properties of Palygorskite: A Comparative Study. Appl. Clay Sci..

[61] Kaufhold S., Dohrmann R., Klinkenberg M., Siegesmund S., Ufer K. (2010). N2-BET Specific Surface Area of Bentonites. J. Colloid Interface Sci..

[62] Budrugeac P. (2020). Critical Study Concerning the Use of Sinusoidal Modulated Thermogravimetric Data for Evaluation of Activation Energy of Heterogeneous Processes. Thermochim. Acta.

[63] Rinnert E., Carteret C., Humbert B., Fragneto-Cusani G., Ramsay J.D.F., Delville A., Robert J.L., Bihannic I., Pelletier M., Michot L.J. (2005). Hydration of a Synthetic Clay with Tetrahedral Charges: A Multidisciplinary Experimental and Numerical Study. J. Phys. Chem. B.

[64] Cases J.M., Bérend I., Besson G., Francois M., Uriot J.P., Thomas F., Poirier J.E. (1992). Mechanism of Adsorption and Desorption of Water Vapor by Homoionic Montmorillonite. 1. The Sodium-Exchanged Form. Langmuir.

[65] Derkowski A., Drits V.A., McCarty D.K. (2012). Rehydration of Dehydrated-Dehydroxylated Smectite in a Low Water Vapor Environment. Am. Mineral..

[66] Bérend I., Cases J.M., François M., Uriot J.P., Michot L., Masion A., Thomas F. (1995). Mechanism of Adsorption and Desorption of Water Vapor by Homoionic Montmorillonites: 2. The Li+, Na+, K+, Rb+, and Cs+-Exchanged Forms. Clays Clay Miner..

[67] Zhang X., He C., Wang L., Li Z., Deng M., Liu J., Li H., Feng Q. (2015). Non-Isothermal Kinetic Analysis of Thermal Decomposition of the Ca-Bentonite from Santai, China. Mineral. Petrol..

[68] Kuang W., Facey G.A., Detellier C. (2004). Dehydration and Rehydration of Palygorskite and the Influence of Water on the Nanopores. Clays Clay Miner..

[69] Bahranowski K., Klimek A., Gaweł A., Serwicka E.M. (2021). Rehydration Driven Na-Activation of Bentonite—Evolution of the Clay Structure and Composition. Materials.

[70] Stackhouse S., Coveney P.V., Benoit D.M. (2004). Density-Functional-Theory-Based Study of the Dehydroxylation Behavior of Aluminous Dioctahedral 2:1 Layer-Type Clay Minerals. J. Phys. Chem. B.

[71] Ogorodova L.P., Kiseleva I.A., Vigasina M.F., Kabalov Y.K., Grishchenko R.O., Mel’Chakova L.V. (2014). Natural Sepiolite: Enthalpies of Dehydration, Dehydroxylation, and Formation Derived from Thermochemical Studies. Am. Mineral..

[72] Li Y., Tian G., Gong L., Chen B., Kong L., Liang J. (2020). Evaluation of Natural Sepiolite Clay as Adsorbents for Aflatoxin B1: A Comparative Study. J. Environ. Chem. Eng..

[73] Jaynes W.F., Zartman R.E., Hudnall W.H. (2007). Aflatoxin B1 Adsorption by Clays from Water and Corn Meal. Appl. Clay Sci..

[74] Xavier K.C.M., Santos M.S.F., Osajima J.A., Luz A.B., Fonseca M.G., Silva Filho E.C. (2016). Thermally Activated Palygorskites as Agents to Clarify Soybean Oil. Appl. Clay Sci..

[75] Gan F., Hang X., Huang Q., Deng Y. (2019). Assessing and Modifying China Bentonites for Aflatoxin Adsorption. Appl. Clay Sci..

[76] Lo Dico G., Croubels S., Carcelén V., Haranczyk M. (2022). Machine Learning-Aided Design of Composite Mycotoxin Detoxifier Material for Animal Feed. Sci. Rep..

[77] Pappas A.C., Tsiplakou E., Georgiadou M., Anagnostopoulos C., Markoglou A.N., Liapis K., Zervas G. (2014). Bentonite Binders in the Presence of Mycotoxins: Results of in Vitro Preliminary Tests and an in Vivo Broiler Trial. Appl. Clay Sci..

[78] Mitchell N.J., Xue K.S., Lin S., Marroquin-Cardona A., Brown K.A., Elmore S.E., Tang L., Romoser A., Gelderblom W.C.A., Wang J.S. (2014). Calcium Montmorillonite Clay Reduces AFB1 and FB1 Biomarkers in Rats Exposed to Single and Co-Exposures of Aflatoxin and Fumonisin. J. Appl. Toxicol..

