# Supplementary material for: Thermogravimetric Analysis of Moisture in Natural and Thermally Treated Clay Materials
Source: Materials (Basel). 2024 May 9;17(10):2231. doi: 10.3390/ma17102231 (PMC11123035; doi:10.3390/ma17102231)
Supplement: Supplementary file 1 [file materials-17-02231-s001.zip › materials-2979389-supplementary.pdf]

Supplementary Materials

Thermogravimetric analysis of moisture in natural and thermally-treated clay materials

Giulia Lo Dico[1,2,3,4], Lorenzo Lisuzzo[4], Verónica Carcelén[3], Giuseppe Cavallaro[4,*], and Maciej Haranczyka[1,*]

[1] IMDEA Materials Institute, C/Eric Kandel 2, 28906 Getafe, Madrid, Spain.
[2] Tolsa Group, Carretera de Madrid a Rivas Jarama, 35, Madrid, Spain. glodico@tolsa.com (G.L.)
[3] Department of Materials Science and Engineering, Universidad Carlos III de Madrid, Avda. de la Unive sidad, 30. 28911 Leganés, Madrid, Spain. vcarcelen@tolsa.com (V.C)
[4] Department of Physics and Chemistry, University of Palermo, Viale delle Scienze, pad. 17, 90128 Palermo, Italy. lorenzo.lisuzzo@unipa.it (L.L.)

* Correspondence: giuseppe.cavallaro@unipa.it (G.C.); maciej.haranczyk@imdea.org (M.H.)

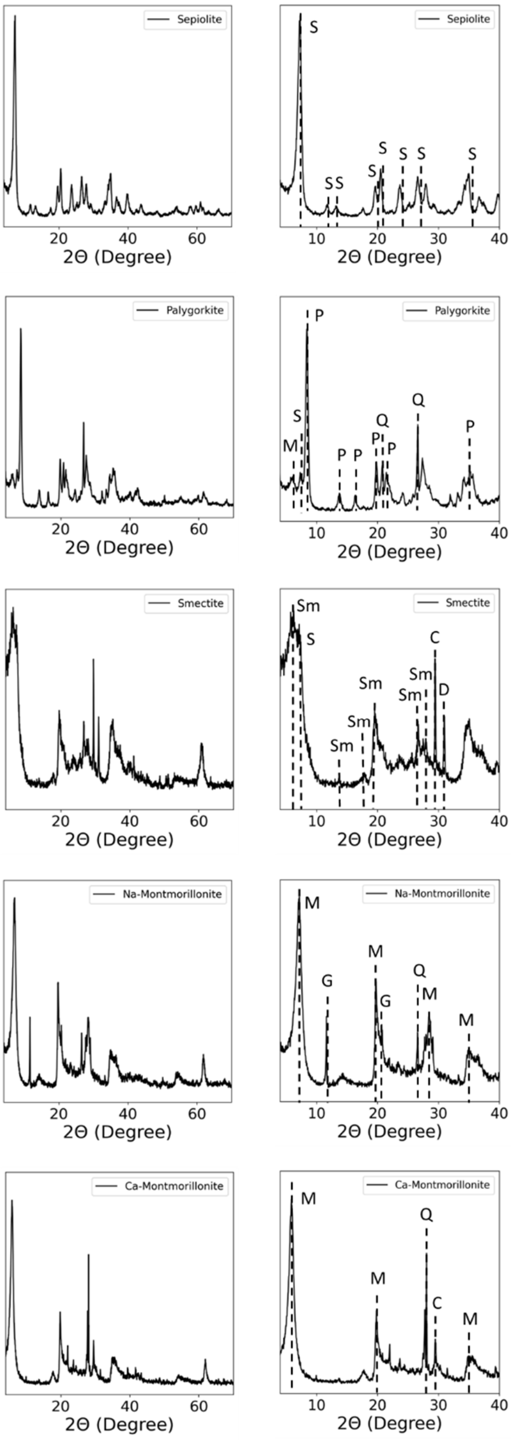

Figure S1. XRD patterns of the studied five samples. The corresponding typical diffraction peaks are highlighted (S=sepiolite, P=paligorkite, Sm=smectite, M=montmorillonite, C=calcite, D=dolomite, Q=quartz).

Table S1. Main crystalline components identified in the XRD patterns.

Sample name	Attapulgite	Sepiolite	Stevensite	Na-montorillonite	Ca-montmorillonite
**Sepiolite (%)**	2	98	10	0	0
**Smectite (%)**	1	0	82	88	90
**Palygorskite (%)**	78	0	0	0	0
**Quartz (%)**	3	0	1	1	1
**Gypsum (%)**	0	0	0	2	0
**Calcite (%)**	0	0	3	0	1
**Dolomite (%)**	0	0	2	0	0
**Total phyllosilicate (%)**	81	98	92	88	90

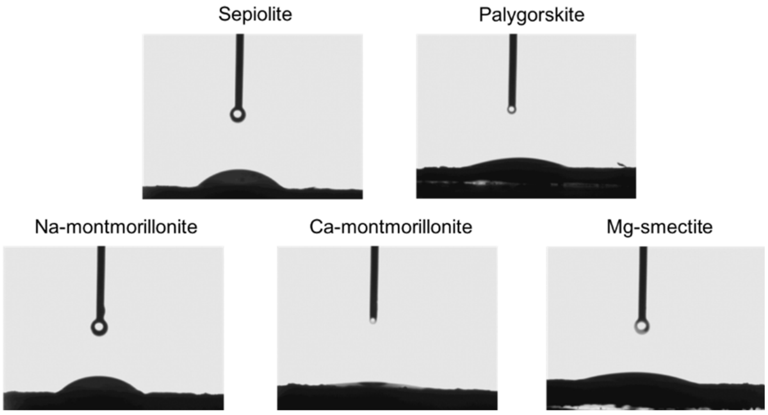

Figure S2. Images of the water droplets just after their deposition on the surface of the five clay samples.

Table S2. β-carotene1 adsorption results for the untreated 5 class of minerals.

Clay minerals	β-carotene ads (%)[a]
Na-Montmorillonite	30
Ca-Montmorillonite	33
Palygorskite	24
Stevensite	35
Sepiolite	29

[a] The amount is expressed as % of the adsorbed molecule with respect to the total starting amount (see Method section for details).
